# Poor Vitamin C Status Late in Pregnancy Is Associated with Increased Risk of Complications in Type 1 Diabetic Women: A Cross-Sectional Study

**DOI:** 10.3390/nu9030186

**Published:** 2017-02-23

**Authors:** Bente Juhl, Finn Friis Lauszus, Jens Lykkesfeldt

**Affiliations:** 1 Medical Department, Aarhus University Hospital, Nørrebrogade 44, 8000 Aarhus C, Denmark; bente311057@gmail.com; 2 Gynecology & Obstetrics Department, Herning Hospital, Gl. Landevej 61, 7400 Herning, Denmark; Finn.Friis.Lauszus@vest.rm.dk; 3 Faculty of Health and Medical Sciences, University of Copenhagen, Ridebanevej 9, Frederiksberg C, 1870 Copenhagen, Denmark

**Keywords:** type 1 diabetes, pregnancy, vitamin C, pregnancy outcome, pregnancy complications, cross-sectional study

## Abstract

Vitamin C (vitC) is essential for normal pregnancy and fetal development and poor vitC status has been related to complications of pregnancy. We have previously shown lower vitC status in diabetic women throughout pregnancy compared to that of non-diabetic controls. Here, we evaluate the relationship between vitC status late in diabetic pregnancy in relation to fetal outcome, complications of pregnancy, diabetic characteristics, and glycemic control based on data of 47 women from the same cohort. We found a significant relationship between the maternal vitC level > or ≤ the 50% percentile of 26.6 μmol/L, respectively, and the umbilical cord blood vitC level (mean (SD)): 101.0 μmol/L (16.6) versus 78.5 μmol/L (27.8), *p* = 0.02; *n* = 12/16), while no relation to birth weight or Apgar score was observed. Diabetic women with complications of pregnancy had significantly lower vitC levels compared to the women without complications (mean (SD): 24.2 μmol/L (10.6) vs. 34.6 μmol/L (14.4), *p* = 0.01; *n* = 19 and 28, respectively) and the subgroup of women (about 28%) characterized by hypovitaminosis C (<23 μmol/L) had an increased relative risk of complications of pregnancy that was 2.4 fold higher than the one found in the group of women with a vitC status above this level (*p* = 0.02, 95% confidence interval 1.2–4.4). No correlation between diabetic characteristics of the pregnant women and vitC status was observed, while a negative association of maternal vitC with HbA1c at delivery was found at regression analysis (*r* = −0.39, *p* < 0.01, *n* = 46). In conclusion, our results may suggest that hypovitaminosis C in diabetic women is associated with increased risk of complications of pregnancy.

## 1. Introduction

The importance of an adequate supply of micronutrients for normal pregnancy and fetal development is well established, particularly in the last trimester due to the increasing needs during the growth spurt of the fetus [1,2]. As early as 1938, Teel and co-workers described the fetus as *acting as a parasite on the mother’s vitamin C pool* based on the observed gradient between maternal plasma and umbilical cord vitamin C (vitC) concentration at term, and the fact that the fetus apparently was preferentially supplied with vitC at the expense of the mother [3,4,5]. Subsequently, several studies have reported that pregnancy in healthy women is associated with a significant decrease in maternal vitC status during pregnancy [4,6,7,8], perhaps partly due to increased blood volume in pregnancy. 

In experimental studies in guinea pigs, which like humans depend on an adequate supply of vitC through their diet, the offspring of vitC deficient guinea pigs have shown abnormalities of fetal bone development, with atrophy of the osteoblasts and retarded osteoid formation [9]. Macroscopic fetal, uterine, and placental hemorrhages as well as poor attachment of the placenta to the uterus were also evident in vitC deficient animals [9]. Other experimental studies have shown an association of infertility, increased incidence of premature- and stillbirths, and increased frequency of abortion with vitC deficiency [10,11]. Intrauterine growth retardation was related to insufficient vitC status in guinea pigs [10]. More recently, experimental reports from animal studies demonstrated that CNS development in particular requires high amounts of vitC and may be impaired by an inadequate maternal supply [12,13,14,15].

In humans, abortion and premature rupture of the fetal membrane are related to low levels of vitC in plasma, leucocytes, and amniotic fluid [16,17,18,19,20,21,22,23,24]. Abnormalities of cardiotocography (CTG) and discolored/green amniotic fluid was also associated with low vitC status at the time of delivery [25]. Furthermore, vitC deficiency may play a leading role in placental abruption [26]. Human studies suggest that poor vitC status leads to fetal oxidative stress and impaired placental implantation due to oxidative stress is thought to increase risk of preeclampsia and miscarriages [27]. Epidemiological studies have also supported an association between vitC deficiency and preeclampsia [28,29]. However recently, human intervention studies using vitC in the prevention of preeclampsia have produced conflicting results [30,31,32]. Another study found no effect of vitamin C on prevention of spontaneous preterm birth [33]. A recent review concluded that a general recommendation of vitC supplementation to pregnant women was not warranted, but subpopulations such as women with vitC deficiency, smokers or diabetics were not discussed [34].

Thus in diabetic animals, experimental data support the amelioration of these risks by vitC supplementation [35,36,37,38]. In one human study, borderline gestational diabetes mellitus had an increased risk of adverse health outcomes compared with women no diabetes [39]. Another human controlled intervention study in type 1 diabetes mellitus (T1DM) pregnancy found a lower risk of premature birth in women receiving vitC and E supplementation and suggested regarding preeclampsia that vitC supplementation may be beneficial in women with a low antioxidant status at baseline; no effect on preeclampsia was observed in the T1DM cohort as a whole [40]. Another study also failed to prevent preeclampsia with vitC and E supplementation in women with T1DM and even a high risk pro-angiogenic haptoglobin genotype [41].

In T1DM, vitC levels are significantly lower than in non-diabetic subjects [42,43]. This seems to be the case in the diabetic pregnancy, too, as we recently reported in a prospective study [8]. We found that the level of vitC was lower throughout pregnancy compared to the control group, and hypovitaminosis C (vitC < 23 μmol/L [44]) was found in 51% of the diabetic women at some stage during pregnancy. Here, we report our evaluation of vitC status in the same cohort of pregnant T1DM women with regard to labor data and the outcome of pregnancies.

## 2. Materials and Methods

All T1DM women from June 1992 to August 1994 attending the Department of Obstetrics, Aarhus University Hospital (Aarhus, Denmark), were screened for participation in the prospective study on vitC during pregnancy and compared to controls as described previously [8]. The inclusion criteria were pregestational T1DM, age >18 years, no other systemic disease than diabetes, and singleton pregnancy. Blood samples for vitC were taken when the diabetic women attended the maternity ward and were taken in a non-fasting state to avoid hypoglycemic episodes. At delivery, an umbilical blood sample for vitC was taken from the newborn. In total, 76 women with T1DM consented to participate in the prospective study [8]. Of these, 47 women had vitC measurements taken in late pregnancy within four weeks of delivery and were included in the present cross-sectional evaluation of vitC status in relation to labor data and outcome of pregnancy. If more than one sample in the 4-week interval before labor were obtained, the mean concentration of the samples was used in the analysis.

Blood samples for plasma vitC measurements were stabilized in sodium EDTA-anticoagulated vacutainer tubes containing dithiothreitol. Tubes were centrifuged and plasma was removed and deproteinized by the addition of 6% perchloric acid. The samples were kept at minus 80 degrees Celsius until analysis and assayed by HPLC using 3,4-dihydroxybenzylamine hydrobromide as internal standard [45]. A plot of the ratio of vitC to internal standard versus the concentration of 6 aqueous standards resulted in a linear curve to at least 86 μmol/L (y = 0.16x − 0.028, R^2^ = 0.99). The within-day and between-day coefficient of variation was 2.6% and 3.9%, respectively, of a mean concentration of 19 μmol/L. Limit of detection and limit of quantification were 0.525 μmol/L and 1.75 μmol/L, respectively. The analytical recoveries were 111%, 104%, 102%, and 101% at vitC concentrations of 5.75, 28.75, 43.125, and 57.5 μmol/L, respectively.

We carried out predefined plasma vitC subgroup analyses according to the 50% percentile of maternal vitC level and these subgroups were used for evaluating other quantitative and qualitative data on pregnancy, labor, and neonates. This 50% percentile was chosen as we a priori had calculated, that we thereby had sufficient data to minimize a type2 error (power > 80%) on expected SDs in relation to third trimester measurements of pregnancy and in relation to labor and fetus related features as we earlier have reported in T1DM pregnancy [8].

Twenty-eight blood samples from the umbilical cords were also obtained as a surrogate measure of the level of vitC of the fetus. However umbilical cord blood was in the same level as found in the heel blood of 200 newborns [25].

The following data were recorded: Age, duration of diabetes, presence of diabetic microangiopathy, glycemic control, diurnal blood pressure, albumin excretion rate, creatinine, creatinine clearance, pregnancy and labor data, and the neonate’s Apgar score at one minute, birth weight, and presence of malformations. The study was part of an evaluation of morbidity in diabetic pregnancy with respect to nephropathy and retinopathy approved by the local Ethics Committee (jr.nr.1992/2523, 1998/4147, and 2026-99). It was performed in concordance with the Helsinki II declaration and all women had given their informed consent. The collection of samples for vitC was approved by the local Ethics Committee (jr.nr. 1992/2328). Hypovitaminosis C was defined as a plasma vitC <23 μmol/L [44].

Preeclampsia was defined as systolic/diastolic blood pressure >140/90 mmHg when normo-hypertensive before week 20 and, simultaneously, albuminuria >300 mg in previously normo-albuminuric women. Pregnancy-induced hypertension was defined as hypertension without signs of preeclampsia. Preterm delivery was defined as delivery following <37 weeks of gestation.

Statistics was performed with IBM SPSS Statistics 20. Difference between two means was tested with Student’s *t*-test if data followed Gaussian distribution; otherwise, Mann-Whitney’s test was used. Proportional data were analyzed by χ^2^ test or Fisher’s Exact test. Values are given as mean ± SD if not otherwise stated. Median (25%–75% interval) indicates variable of non-Gaussian distribution and values were subjected to non-parametric testing. A two-sided *p* < 0.05 was chosen as level of significance.

## 3. Results

Clinical data from the pregnant diabetic women are shown in Table 1 and are also presented in subgroups according to the median value (25%–75%) of maternal plasma vitC taken within four weeks of delivery. All comparisons of baseline data and diabetic characteristics in relation to the 50% percentile of vitC (26.6 (22.0–37.2) μmol/L) were non-significant (Table 1). The range (0%–100%) of plasma vitC in the cohort was 3.1–61.0 μmol/L.

Results regarding pregnancy and fetal related features are presented in Table 2. No relationship between maternal vitC level and birth weight or Apgar score was observed. Nor was the way of delivery (acute cesarean section, elective cesarean and induced delivery; 7/19/21) associated with vitC status. Moreover, no difference was observed in the level of HbA1c in relation of the median maternal vitC of 26.6 μmol/L, but a negative association of maternal vitC with HbA1c at delivery was found at regression analysis (*r* = −0.39, *p* = 0.006, *n* = 46). The vitC levels of the umbilical cord blood correlated positively with the obtained Apgar score of the newborn (*r* = 0.45, *p* = 0.011), also when corrected for maternal vitC, HbA1c and diabetes duration (*r* = 0.52, *p* = 0.025).

Hypovitaminosis C was found in 13 out of 47 diabetic women (28%) and was associated with a risk of complications of 69%, while the risk of complications was 29% in case of higher levels of vitC (Table 3). The relative risk of having complications of pregnancy was 2.4 times in case of maternal hypovitaminosis C compared to higher levels of maternal vitC (*p* = 0.02). In accordance, the diabetic women with complications of pregnancy had a significantly lower vitC status in late pregnancy compared to those without complications (mean (SD) 24.2 μmol/L (95% CI: 19.4–30) vs. 34.6 μmol/L (95% CI: 29.6–40); *p* = 0.011, *n* = 19 and 28, respectively). The type and distribution of complications are given in Table 4.

## 4. Discussion

The present cross-sectional study of T1DM pregnancy found an inverse relationship between vitC status and risks of complications in pregnancy. Thus, poor vitC status within four weeks of delivery was a positive predictor (69%) for complications of pregnancy, while a maternal vitC >23 μmol/L was a negative predictor (71%) for complications of pregnancy, respectively. In support of the observed relationship between maternal vitC status in late pregnancy and complications, we found a low maternal plasma vitC in case of complications of pregnancy (power of test > 80%).

The mean level of vitC was 24.2 μmol/L in the group with complications in pregnancy, thus in this normally distributed group nearly the half of the women had a level of vitC characterized as hypovitaminosis C. Much of the literature showing associations between vitC status and complications in pregnancy was conducted in pregnant experimental animals with or without induced diabetes and related to severe vitC deficiency (<11 μmol/L). This level increases the risk of developing outright scurvy, the ultimately mortal manifestation of prolonged severe vitC deficiency. However, only about 4% of the present cohort (2 patients out of 47) had severe vitC deficiency within four weeks of delivery and no clinical symptoms of scurvy were recorded in the case records of the pregnant women in this study. Therefore, it appears that the complications in diabetic pregnancy are already present at suboptimal vitC levels. In agreement, previous human studies identified a range of complications of pregnancy in non-diabetic women, the risks of which were inversely correlated with plasma vitC; this was, indeed, found over a wide concentration range above the level critical for development of scorbutic manifestations [16,17,18,19,20,21,22,23,24,25,26,27]. Thus, although higher levels of vitC are not associated with scurvy, lack of scurvy does not preclude the presence of several other negative health effects of a suboptimal vitC status, and the optimal vitC intake in humans is still a matter of considerable debate [46]. 

In humans, a randomized placebo-controlled intervention study with vitamin C and E in T1DM pregnancies showed no overall effect of supplementation (1000 mg vitamin C and 400 IU vitamin E (α-tocopherol) daily until delivery) on the incidence of preeclampsia [40]. However, subgroup analysis did reveal a significant positive effect of supplementation vs placebo on preeclampsia among patients who were vitC deficient at baseline (<10 μmol/L). Thus, the authors suggested that the significant benefit of supplementation on preeclampsia may be limited to women with severe vitC deficiency [40]. VitC and E supplementation also resulted in fewer preterm deliveries compared to placebo in the cohort as a whole, but the potential correlation to vitC status at entry was not explored [40]. Another study has also reported lack of effects of supplementation with vitC on the incidence of preeclampsia in high-risk T1DM women [41]. The absence of effect of vitC supplementation on preeclampsia in humans with or without diabetes may arise from the variation in the degree of plasma saturation and subsequent differential outcomes of supplementation as discussed elsewhere [47].

Another interesting result of the present study is the difference in vitC level in umbilical cord blood of newborns reflects some of the difference in the mothers’ vitC level. Combined with the observation that the ratio of umbilical cord/maternal vitC favors babies born by mothers with vitC level below the median, our data collectively support the notion that the fetus is preferentially supplied with vitC at the expense of the mother [5,48]. However, as the vitC level in these babies is significantly lower than that of those born by mothers with vitC level above the median, it also suggests that such a preferential supply cannot fully compensate for poor maternal vitC status. The maternal as well the umbilical vitC measurements were conducted with sufficient data to minimize a type 2 error on conclusions (power of *t* test > 80%). Thus in this study—in spite of the fetus acting as a “parasite” as described by Teel et al. [3]—the newborns of mothers with low maternal vitC seem not to be able to obtain the same level of vitC in the umbilical cord as newborns of mothers with a higher vitC level, although their ratio is larger. This is in line with experimental data from guinea pigs showing that the preferential fetal transport may be overridden by increased needs of the mother during situations of deficiency, thereby potentially influencing the health of the offspring [13,49]. In accordance, the vitC levels of the umbilical cord blood correlated positively with the obtained Apgar score of the newborn.

Finally, no correlation between diabetic characteristics of the pregnant women and vitC status was observed, although glycemic control measured as HbA1c showed an inverse correlation with maternal vitC level. VitC is thought to be actively transported by SVCT transporters in the placenta [50]; however, it also shares the same transporters as glucose via the GLUT-mediated transport of dehydroascorbic acid (DHA; the oxidized form of vitC) [51]. Thus, it may be speculated that the degree of glycemic control and, consequently, the level of oxidative stress and ascorbate oxidation rate may affect the bioavailability of vitC in T1DM pregnant women through competitive inhibition of DHA transport as proposed by Mann and Newton already in 1975 [52] and supported by the NHANES study 2003–2006 data [53]; here an inverse relationship between vitC and HbA1c was reported in 7697 non-diabetic participants. Moreover, Tu et al. have recently proposed that impaired red cell recycling of DHA may be a key link in diabetes [54]. 

Limitations of the present study include the small number of participants and that the registration of complications of pregnancy was done retrospectively on the case report forms, which in some cases may be imprecise. The included T1DM patients with diabetic complications, i.e., retino- and nephropathy, could potentially influence the outcome of pregnancy. However, we did not find any relationship of these variables with vitC probably due to the small number of participants. Finally, the samples for vitC were taken in a non-fasting state to avoid hypoglycemic episodes, which may have increased the SD of the vitC measurements and, thus, the risk of type 2 error. 

## 5. Conclusions

In conclusion, the results from this small study of a pregnant T1DM cohort suggest that hypovitaminosis C in late pregnancy may be associated with an increased risk of developing complications in pregnancy and may also, to some extent, limit the obtainable level of vitC of the fetus as measured by umbilical values in the newborn. Further investigations are needed to disclose the possible clinical significance of vitC in the diabetic pregnancy and to confirm in larger studies that a benefit of vitC supplementation exists in pregnancies characterized by hypovitaminosis C.

## Figures and Tables

**Table 1:** Clinical data and characteristics of the diabetic status by maternal vitamin C (vitC) within the last four weeks of pregnancy (*n* = 23/24) and of the whole cohort (*n* = 47).

	VitC > Median >26.6 μmol/L	VitC ≤ Median ≤26.6 μmol/L	*p* Value	Characteristics of the Whole Cohort
Vit C (μmol/L), *n* = 23/24/47	37.1 (28.2–61.0) ^1^	22.1 (3.1–28.2)		30.1 (13.6)
Age (yr), *n* = 23/24/47	28.8 (3.7) ^2^	27.7 (3.5)	0.314	27 (26–31)
Maternal weight at delivery (kg) *n* = 12/11/23	78.5 (72.3–87.5)	74.0 (68.0–86.0)	0.32	78 (70–86)
Maternal height (cm), *n* = 11/11/22	166.2 (6.2)	164.4 (8.3)	0.569	165.3 (7.2)
Diabetes duration (year), *n* = 23/24/47	15.0 (8.9)	13.2 (9.0)	0.486	14.1 (8.9)
Parity, *n* = 23/24/47	1.8 (0.8)	1.8 (0.7)	0.876	2 (1.2)
Systolic blood pressure at entry (mmHg), *n* = 15/14/29	120.1 (10.3)	120.5 (9.9)	0.641	120.0 (9.9)
Diastolic blood pressure at entry (mmHg) *n* = 15/14/29	72.1 (6.6)	70.9 (6.7)	0.673	71.2 (6.9)
Retinopathy Non/Simplex/Proliferative, *n* = N/S/P	12/8/3	12/9/3	0.881	24/17/6
BMI (kg/m^2^) at delivery, *n* = 11/10/21	29.2 (3.8)	27.6 (4.4)	0.607	28.6 (4.3)
Normo-/Micro-/Macro-albuminuria *n* = N/Mi/Ma	18/4/1	20/4/0	0.581	38/8/1
HbA1c (%) at entry, *n* = 22/23/45	7.7 (1.6)	7.9 (1.2)	0.0697	7.7 (1.4)
Creatinine clearance at entry (ml/min) *n* = 15/15/30	123.3 (22.1)	116.2 (32.3)	0.869	122.1 (27.1)
Smoking, *n* = Yes/no/unknown	6/16/1	10/14/0	0.538	16/30/1

^1^ VitC levels in each subgroup is reported given as median (range); ^2^ Other data are listed as mean (SD), median (25%–75%) or *n*-values.

**Table 2:** Labor and fetus related features in relation to above or below the median level of maternal vitC in late pregnancy.

	VitC > Median >26.6 μmol/L	VitC ≤ Median ≤26.6 μmol/L	*p* Value
VitC in umbilical cord (μmol/L), *n* = 12/16	101.0 (16.6) ^1^	78.5 (27.8)	0.02
Umbilical cord/maternal vitC ratio, *n* = 12/16	2.6 (2.1–2.9)	4.1 (2.8–5.1)	0.007
Apgar score at one minute, *n* = 19/23	10 (9–10)	9 (9–10)	0.56
Birth weight (g), *n* = 23/24	3867 (649)	3533 (771)	0.12
Gestations age at labor (weeks), *n* = 23/24	37.4 (1.1)	37.2 (1.5)	0.64
Normal delivery (*n*)	0	0	
Induced delivery and elective section/acute section, (*n*/*n*)	20/3	20/4	1.0
HbA1c at delivery (%), *n* = 23/23	6.7 (1.1)	7.2 (1.0)	0.14

^1^ Data are listed as mean (SD), median (25%–75%) or *n*-values.

**Table 3:** Women with complications in subgroups according to vitC status in late pregnancy.

Complications of Pregnancy	Hypovitaminosis C ^1^	Above Hypovitaminosis C Level	All Women	Fisher‘s Exact Test
Yes (*n*)	9	10	19	
No (*n*)	4	24	28	
Total (*n*)	13	34	47	*p* = 0.02

^1^ Plasma concentration <23 μmol/L.

**Table 4:** The type and distribution of complications in T1DM women (*n* = 47). Recorded complications were prematurity, gestational hypertension, asphyxia, malformation, still birth, placental abruption, preeclampsia.

Complication	Frequency	VitC μmol/L Mean (SD)
Women with/without complications	19/47 vs. 28/47	24.2 (10.6)/34.6 (14.4)
Fetal malformation	4/47 ^1^	18.1 (9.0)
Asphyxia/abnormal CTG ^2^	9/47	22.9 (12.8)
Preeclampsia	5/47	25.0 (10.6)
Prematurity	5/47	20.9 (6.0)
Placental abruption	2/47	18.6 (0.9)
Still birth	2/47	30.9 (0.4)
Pregnancy-induced hypertension	2/47	30.0 (7.1)

^1^ The fetal malformations consisted of two neonates with cardiac malformations with transposition and atrium septum defect and two others were related to skeletal abnormalities; ^2^ CTG: Cardiotocography. Abnormal CTG was diagnosed in nine women at delivery and of these, seven ended in acute cesarean section and two in induced delivery. Women may have more than one complication.
